# Immune Response to BNT162b2 mRNA COVID-19 Vaccine in a Cohort of Healthcare Workers

**DOI:** 10.7759/cureus.75188

**Published:** 2024-12-05

**Authors:** Gemma Grau Gómez, Xavier Martínez Lacasa, Angeles Jaen, Judith Vidal Martínez, Emma Padilla, David Clemente, Siena Molina, Ales Chlouba, Susana González, Helena Monzón Camps

**Affiliations:** 1 Internal Medicine, Fundació Assistencial Mútua Terrassa, Terrassa, ESP; 2 Research Unit, Fundació, Docència i Recerca Mútua Terrassa, Terrassa, ESP; 3 Cytometry Department, Catlab, Fundació Assistencial Mútua Terrassa, Terrassa, ESP; 4 Microbiology Department, Catlab, Fundació Assistencial Mútua Terrassa, Terrassa, ESP; 5 Occupational Health, Fundació Assistencial Mútua Terrassa, Terrassa, ESP

**Keywords:** covid-19, humoral response, lymphocyte subpopulations, sars-cov-2, vaccination

## Abstract

In this 13-month follow-up study, we examined a cohort of vaccinated healthcare workers without prior SARS-CoV-2 infection to assess the humoral and cellular response to the BNT162b2 mRNA COVID-19 vaccine over time. We measured median immunoglobulin G and lymphocyte subpopulation levels after the first and second doses, at five months post-second dose, and before and after the third dose. Our findings evinced a remarkable initial cellular and humoral response to each vaccine dose, although a progressive decline suggests the potential need for long-term booster doses. Age analysis showed significantly higher immunoglobulin G levels in the younger group after the first dose administration, although these differences were not maintained in the following doses. Preserving cellular immunity could assure long-lasting protection against SARS-CoV-2 infection.

## Introduction

The severe acute respiratory coronavirus 2 (SARS-CoV-2) pandemic syndrome has entailed an unprecedented scenario regarding the fast development of safe and effective vaccines. The common goal that all vaccines pursue is to activate the immune system against the pathogen and generate cellular memory, mimicking the infection, but avoiding the harmful effects of it. This is achieved by stimulating both innate and adaptive immunity through adjuvants and antigens contained in the vaccine. Surface proteins of the virus activate immature CD19+ B lymphocytes, which differentiate into plasmatic cells producing antibodies to destroy infected cells, or into memory cells for future recognition of the antigen. While humoral response only represents a part of this immune activation, its widespread use and standardization allow it to be studied easily [1]. On the other hand, assessment of cellular response provides insight into individual immune response to the vaccine and its relation to humoral immunity. In the case of SARS-CoV-2, given the high incidence of infection despite several lockdowns, mass vaccination was believed to be the best option to try to control the pandemic. In this regard, the first vaccines including the mRNA vaccine from BioNTech/Pfizer (BNT162b2) and the vector-based vaccine by AstraZeneca (Vaxzevria) were approved worldwide in December 2020. Both vaccines needed a primary dose and a booster dose administered within several weeks, typically three weeks for the BioNTech/Pfizer vaccine, and eight to 12 weeks for the AstraZeneca vaccine. In both cases, the vaccine induced an immune response against the S1 spike protein of the virus [2], and initial studies evinced high effectiveness in preventing not only the symptoms in case of infection, but also virus transmission [3-5]. However, individual immune responses varied depending on personal factors such as age-related immune system decline in the elderly [6,7], as had been previously reported regarding other pathogens [8]. In addition, some studies have also shown a correlation between a decreased immune response to vaccination and smoking [9] as well as immunosuppression [10]. healthcare workers (HCWs) have been key in the fight against the COVID-19 pandemic by providing care to affected patients. Recognizing their high exposure to the virus in the workplace and close contact with vulnerable populations, HCWs were prioritized for COVID-19 vaccination. Consequently, understanding their immune response to SARS-CoV-2 vaccines and their long-term dynamics has garnered significant interest. Studies have shown a remarkable humoral response, particularly following two doses of BNT162b2 mRNA COVID-19 vaccine [9] along with high short-term protection against SARS-CoV-2 infection [11]. In the current study, we present a follow-up analysis of a group of HCWs in an acute care hospital who were not previously infected with SARS-CoV-2, focusing on their vaccination status. The aim of the study is to assess the dynamics of lymphocyte subpopulations and humoral response to the BNT162b2 mRNA COVID-19 vaccine over time.

## Materials and methods

Study design

This prospective observational study followed a cohort of 149 vaccinated HCWs in Spain for 13 months. The study has been reported as per the STROBE (Strengthening the Reporting of Observational Studies in Epidemiology) guidelines [12]. Estimating an expected effectiveness ratio of 90%, based on clinical trials demonstrating vaccine efficacy around 95% [13], we calculated the sample size to achieve a ±5% precision with a 5% alpha error and bilateral contrast. The target population comprised 1.760 HCWs from an acute care teaching hospital, factoring in an estimated 15% loss to follow-up and a 10% replacement rate to account for subjects not meeting inclusion criteria.

Considering these factors, a total of 164 HCWs were initially selected by simple randomization using the Epidat 3.0 program (Galsoft Linux, Spain). From this first selection, 15 subjects with a history of COVID-19 infection or previous serology indicating the presence of antibodies against SARS-CoV-2 were excluded from the study. This resulted in a final sample of 149 participants for the analysis.

Subjects’ selection

Subjects included in the study were HCWs from an acute care teaching hospital, with no record of previous COVID-19 infection or positive serology against SARS-CoV-2, who had received the first dose of the BNT162b2 vaccine. These HCWs were willing to provide written informed consent prior to the first blood sample extraction and verified the absence of infection at baseline and follow-up visits through real-time reverse-transcriptase polymerase chain reaction (RT-PCR) or rapid antigen testing on nasopharyngeal swabs, depending on test availability. Therefore, exclusion criteria were age under 18 years old, previous history of COVID-19 infection, or positive serology indicating the presence of antibodies against SARS-CoV-2 and/or positive results from RT-PCR or rapid antigen testing for SARS-CoV-2 prior to each blood sample extraction.

Study variables

Independent variables included age, gender, and professional category. Dependent variables were, on one hand, quantitative immunoglobulin G (IgG) expressed as AU/mL and a dichotomous IgG response (positive IgG response: Yes/No); and on the other hand, quantification of lymphocyte subpopulations expressed as cells/µL, including CD3+ total T lymphocytes, CD3+CD4+ T helper lymphocytes, CD3+CD8+ T cytotoxic lymphocytes, CD19+ B lymphocytes and CD56+CD16+ natural killer (NK) lymphocytes.

Information regarding independent variables was collected through a questionnaire completed by participants before vaccination. IgG levels were obtained using an anti-spike IgG chemiluminescent semiquantitative immunoassay technique (LIAISON® SARS-CoV-2 TrimericS IgG; DiaSorin, Stillwater, MN, USA), with a value over 12 AU/ml considered a positive IgG response. Lymphocyte subpopulation quantification was conducted via flow cytometry (BD FACSCantoTM II Clinical Flow Cytometry System) using a standardized protocol with a single platform.

Samples collection and follow-up

Blood samples were collected from the 149 HCWs included in the study to analyze lymphocyte subpopulations and IgG response to the vaccine over the study period. Prior to each blood sample collection, RT-PCR tests or rapid antigen tests on nasopharyngeal swabs were carried out to exclude subjects with SARS-CoV-2 infection.

The immune response follow-up was conducted at different stages: the first blood sample was obtained at 19 ± 3 days after administration of the first vaccine dose; the second blood sample was collected 43 ± 2 days after the administration of the second dose; the third blood sample was performed at 156 (5 months) ± 7 days after the administration of the second dose; the fourth blood sample was taken 46 days ± 6 days before the administration of the third dose; finally, the fifth blood sample was acquired 65 days ± 7 days after the administration of the third dose.

**Figure 1:**
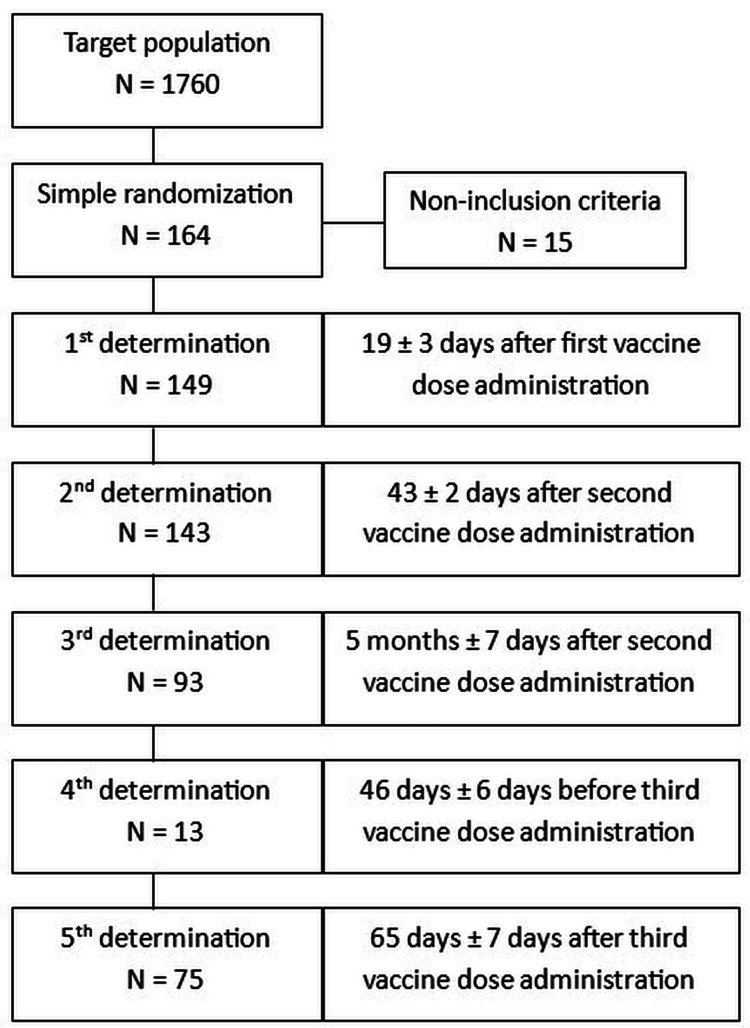
Sample size in each phase of the study. In fourth determination there is an important decrease in the number of subjects due to the need for rapid vaccination.

The sample size across all the phases of the study is represented in the following algorithm (Figure 1).

Analytical procedures

To conduct a comprehensive study of cellular immunity, it is necessary to determine lymphocyte subpopulations in whole blood samples with the anticoagulant ethylenediamine tetraacetic acid (EDTA) and to perform an activation and senescence profile of T and B memory lymphocytes after stimulation with SARS-CoV-2 peptide pools. However, the latter was not carried out in our study due to the need for internal validation of a non-standardized method with specific technical requirements that were not feasible to meet due to high workload constraints at the time. Therefore, our study focused solely on the quantification of circulating lymphocyte subpopulations using flow cytometry without prior stimulation.

Regarding immunoglobulin quantification, it is important to note that the cut-off point for the immunoassay technique was changed to 791 AU/ml during the study. This adjustment was made following a correlation of results by the provider (DiaSorin, Stillwater, MN, USA) with the units and values established in the first World Health Organization (WHO) International Standard for anti-SARS-CoV-2 immunoglobulins. The aim was to standardize techniques across different commercial suppliers in order to compare the results.

Statistical analysis

All statistical analyses were performed using Stata S/E versions 17.0 and 18.0 (StataCorp LLC, College Station, TX, US). Quantitative variables were expressed as mean (standard deviation {SD}) or median (interquartile range {IQR} 25-75%), as appropriate. Qualitative variables were expressed as proportions with 95% confidence intervals (CI95). The comparison of median IgG values and median lymphocyte subpopulation values at each blood sample determination during follow-up was performed using the Wilcoxon signed-rank test for pairwise comparisons and the Friedman test for multiple comparisons. In addition, median IgG values and median lymphocytic subpopulation values for each age group (18-30 years, 31-60 years, >60 years) and determination were analyzed using Kruskal-Wallis tests. Furthermore, the percentage and CI95 of positive IgG response (as a qualitative dependent dichotomous variable yes/no) were calculated at baseline and defined at each follow-up time point. Bivariate analyses between positive IgG response and age (both quantitative and qualitative-grouping of age), gender, and professional category were conducted using the Mann-Whitney U test or Kruskal-Wallis test, as appropriate. Finally, the correlation between IgG values and different lymphocyte subpopulation values was determined using Spearman's or Kendall's tests, as appropriate, for each determination. A p-value <0.05 was considered statistically significant.

Ethical clearance

Written informed consent was obtained from all participants and recorded in their medical history. The study was approved by the Ethics Committee for Research With Medicines of the Fundació Assistencial Mútua Terrassa (approval number: P/24-129, dated November 27, 2024).

## Results

Humoral response

The first blood sample was obtained at 19 ± 3 days after administration of the first dose of BNT162b2 mRNA COVID-19 vaccine in a total of 149 HCWs. Among them, 111 were women (74.5%) and the average age of the cohort was 41 years ± 12. The distribution by professional category was: 27.1% physicians, 33% nurses, 11.8% clinic assistants, and 28% other personnel. In this determination, 145 subjects (97.3%; CI95: 93.3-99.3) presented a positive IgG response against SARS-CoV-2, with a median (IQR 25-75%) IgG level (AU/mL) of 181 (99-298), with only one subject (0.7%) showing an IgG quantification superior to 800. There were 4 subjects who were seronegative. 

The second blood sample was collected 43 ± 2 days after the administration of the second vaccine dose in a total of 143 participants, as six subjects (two women and four men) were lost to follow-up. The average age of this group was 42 ± 12. All participants in this group presented a positive IgG determination (100%), with a median IgG level of 735 (564-801). Additionally, 65 subjects (45.5%) exhibited an IgG quantification superior to 800.

The third blood sample was collected 156 (5 months) ± 7 days after the administration of the second vaccine dose in a total of 93 HCWs. Among them, 66 (70.9%) were men, with an average age of 43 ± 12. Results from this sample revealed median IgG levels of 223 (162-305). None of the subjects showed an IgG quantification superior to 800.

Statistical analysis revealed differences in median values of IgG quantification before and after the second dose, indicating a significant increase in IgG levels after the second dose administration (p-value < 0.001). Statistical analysis also showed differences in median values of IgG quantification when comparing the first and third determinations, with a significant increase in IgG levels after the third determination (p-value < 0.001).

The fourth blood sample was taken 355 ± 5 days after the second dose administration and 196 ± 8 days after the third determination in 13 subjects (9 women, 69%). In this determination, all subjects (100%) presented a positive IgG response against SARS-CoV-2, with median IgG levels of 90 (52.5-154).

The fifth blood sample was collected 65 days ± 7 days after the administration of the third dose administration in a total of 75 subjects (59 women, 78.7%). The cut-off point of the technique had already been changed to 791. In this final determination, all participants (100%) presented a positive IgG response against SARS-CoV-2 of 791 or above. 

**Figure 2:**
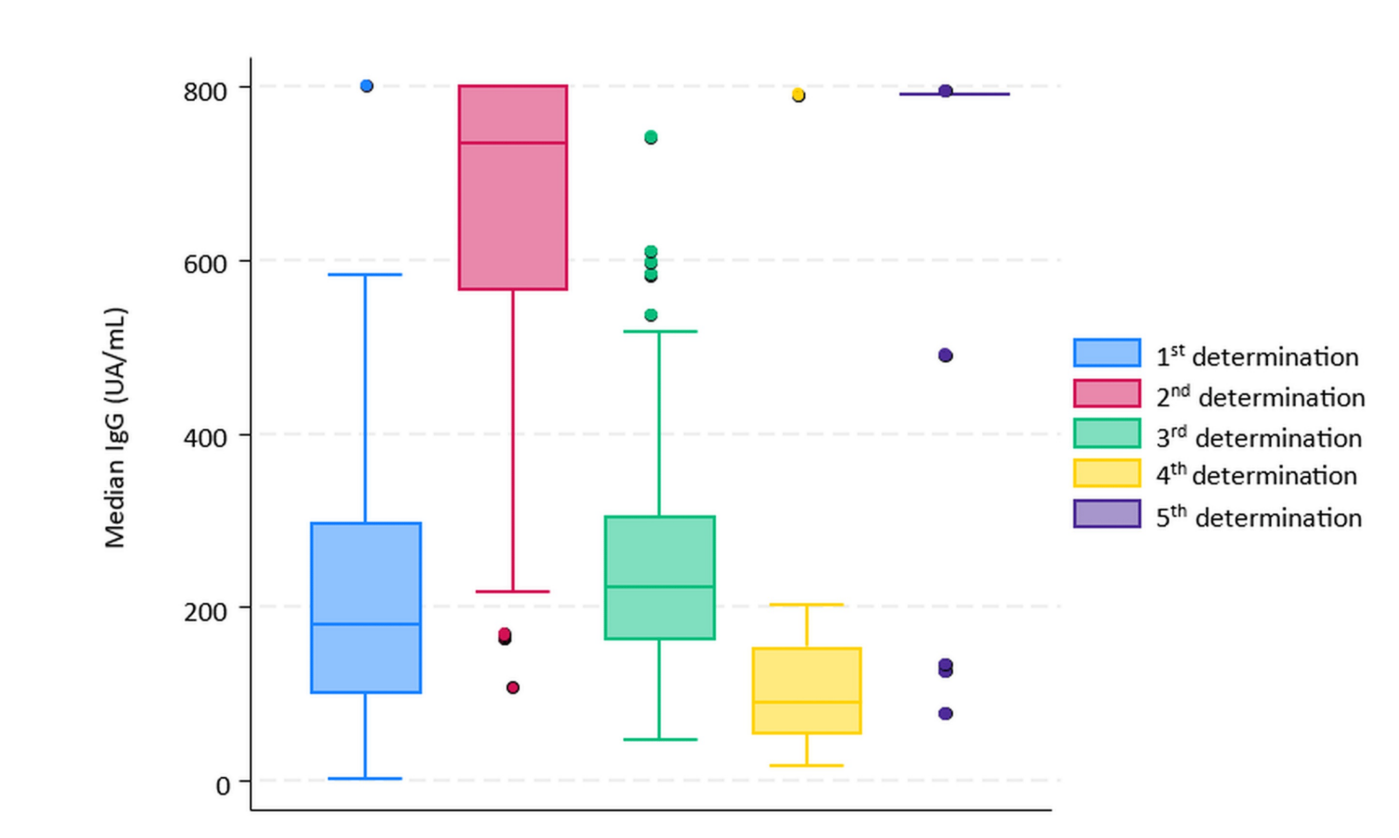
Median comparison of IgG values for all determinations. First determination presented median (IQR 25-75%) IgG level (AU/mL) of 181 (99-298), second determination of 735 (564-801), third determination of 223 (162-305), fourth determination of 90 (52.5-154) and fifth determination of 90 (52.5-154).

Comparison of median IgG values for each of the five determinations showed statistically significant differences (p=0.0341). Median IgG values throughout follow-up (Figure 2).

In addition, complementary analysis was conducted to compare IgG values among different age groups, with participants divided into three categories: 18 to 30 years (n=36; median age 25, IQR 24-28); 31 to 60 years (n=103; median age 44, IQR 39-52) and over 60 years (n=10; median age 62, IQR 61-63). Following the first vaccine dose, the median IgG value was found to be higher in the 18 to 30 years age group, while the lowest value was observed within the group over 60 years, emerging a statistically significant difference (p=0.0001). However, despite observing a tendency towards lower median IgG values in the oldest group after the second dose administration, the difference was not statistically significant (p=0.1615). Furthermore, no significant differences were observed in the third (p=0.9820), fourth (p=0.6143), and fifth (p=0.8240) determinations regarding immune response between age groups.

Within each age group, a comparison was conducted between median IgG values from the five determinations, and no statistically significant differences were observed. Finally, there were no significant differences found when analyzing median IgG values related to gender (p=0.45) or professional category (p=0.54).

Lymphocyte subpopulations

The first determination was performed at 19 ± 3 days after the administration of the first dose of the BNT162b2 mRNA COVID-19 vaccine in 143 individuals. The analysis revealed the following median levels (expressed in cells/µL) of lymphocytes subpopulations: CD3+ T lymphocytes levels of 1620 (1310-1920), CD3+CD4+ T helper lymphocytes levels of 1010 (770-1260), CD3+CD8+ T cytotoxic lymphocytes levels of 560 (420-760), CD19+ B lymphocytes levels of 230 (160-330) and CD56+CD16+ NK lymphocytes levels of 310 (210-400).

As regards the second determination, data was collected at 43 ± 2 days after receiving the second vaccine dose from 143 subjects. The analyses revealed median levels (expressed in cells/µL) of lymphocyte subpopulation as follows: CD3+ T lymphocytes levels of 1630 (1300-2010), CD3+CD4+ T helper lymphocytes levels of 990 (820-1240), CD3+CD8+ T cytotoxic lymphocytes levels of 570 (410-750), CD19+ B lymphocytes levels of 230 (160-300) and CD56+CD16+ NK lymphocytes levels of 330 (250-460).

The third determination was conducted at 156 ± 7 days after receiving the second vaccine dose in 84 subjects. Analysis evinced median levels (expressed in cells/µL) of lymphocyte subpopulations as follows: CD3+ T lymphocytes levels of 1520 (1240-1830), CD3+CD4+ T helper lymphocytes levels of 960 (760-1170), median CD3+CD8+ T cytotoxic lymphocytes levels of 560 (430-730), CD19+ B lymphocytes levels of 230 (170-320) and CD56+CD16+ NK lymphocytes levels of 290 (210-370).

The fourth determination was conducted approximately 10 months after the administration of the second vaccine dose and 46 ± 6 days before receiving the third dose in 12 participants. The results denoted median levels (expressed in cells/µL) of lymphocyte subpopulations as follows: CD3+ T lymphocytes levels of 1330 (1100-1710), CD3+CD4+ T helper lymphocytes levels of 860 (660-1130), CD3+CD8+ T cytotoxic lymphocytes levels of 450 (390-540), CD19+ B lymphocytes levels of 180 (100-270) and CD56+CD16+ NK lymphocytes levels of 260 (180-380).

**Figure 3:**
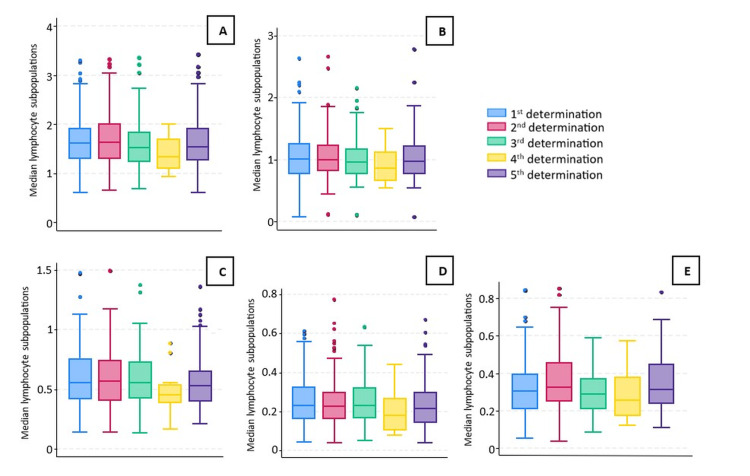
Median lymphocyte subpopulations levels in all five determinations; (A) median CD3+ T lymphocytes; (B) median CD3+CD4+ T helper lymphocytes; (C) median CD3+CD8+ T cytotoxic lymphocytes; (D) median CD19+ B lymphocytes; (E) median CD56+CD16+ NK lymphocytes. (A) Median CD3+ T lymphocytes: first determination 1620 (1310-1920), second determination 1630 (1300-2010), third determination 1520 (1240-1830), fourth determination 1330 (1100-1710) and fifth determination 1530 (1270-1920). (B) Median CD3+CD4+ T helper lymphocytes: first determination 1010 (770-1260), second determination 990 (820-1240), third determination 960 (760-1170), fourth determination 860 (660-1130) and fifth determination 970 (770-1220). (C) Median CD3+CD8+ T cytotoxic lymphocytes: first determination 560 (420-760), second determination 570 (410-750), third determination 560 (430-730), fourth determination 450 (390-540), and fifth determination 530 (400-650). (D) Median CD19+ B lymphocytes: first determination 230 (160-330), second determination 230 (160-300), third determination 230 (170-320), fourth determination 180 (100-270), and fifth determination 210 (140-300). (E) Median CD56+CD16+ NK lymphocytes: first determination 310 (210-400), second determination 330 (250-460), third determination 290 (210-370), fourth determination 260 (180-380) and fifth determination 310 (240-450).

Finally, the fifth determination was conducted 65 ± 7 days after the administration of the third vaccine dose in 76 subjects. The analysis revealed median levels (expressed in cells/µL) of lymphocyte subpopulations as follows: CD3+ T lymphocytes levels of 1530 (1270-1920), CD3+CD4+ T helper lymphocytes levels of 970 (770-1220), CD3+CD8+ T cytotoxic lymphocytes levels of 530 (400-650), CD19+ B lymphocytes levels of 210 (140-300) and CD56+CD16+ NK lymphocytes levels of 310 (240-450). Median lymphocyte subpopulation levels in all five determinations are depicted in absolute values within the following graphs (Figure 3).

By and large, the observed dynamics throughout the time of all lymphocyte subpopulations were the expected after three vaccine doses and evinced a similar pattern for all subpopulations. It must be said that complementary analyses between lymphocyte subpopulations by age group in each determination were performed, and no statistically significant differences were observed.

Correlation between humoral response and lymphocyte subpopulations

The correlation between humoral response and each lymphocyte subpopulation was analyzed for all five determinations. In the first, second, and third samples, the analysis dismissed any statistically significant correlations. However, in the fifth determination, results revealed statistically significant positive correlations between IgG and CD3+ T lymphocytes, with a Spearman’s rank correlation coefficient (rho) of 0.24 (p=0.04) as well as between IgG and CD3+CD8+ T cytotoxic lymphocytes, with a Spearman’s rho of 0.28 (p=0.01). Additionally, in the fifth determination, there was a non-significant tendency towards a positive correlation between IgG and CD3+CD4+ T helper lymphocytes, with a Spearman’s rho of 0.22 (p=0.06).

## Discussion

The present study aims to characterize the immune response to three doses of the BNT162b2 mRNA COVID-19 vaccine among a cohort of healthcare workers who were not previously infected with SARS-CoV-2. Our study reveals a remarkable cellular and humoral response after each vaccine dose, albeit with a progressive decrease, highlighting the potential need for booster doses. 

The first determination represents the primary response to the vaccine, as it marks the initial exposure to the antigen. During this phase, the subject’s innate immunity is activated, leading to the subsequent induction of adaptative immunity [14]. This initial response triggers antibody production by plasmatic cells, which gradually decreases as the antigen is eliminated. However, this primary response may not provide enough protection against future SARS-CoV-2 infections, making necessary the administration of a second vaccine dose. In contrast, the second determination represents a secondary response to the antigen. By this stage, the immune system has developed specific memory cells, including T and B memory lymphocytes. These memory cells enable a quicker and more efficient response upon re-exposure to the antigen, resulting in a more robust and long-lasting antibody production. The present study reveals a generally mild increase in lymphocyte subpopulation levels, although not statistically significant. Notably, there is a decrease in B lymphocytes, aligning with the peak of IgG production, as described in the literature [15]. Nevertheless, the absence or scarcity of memory cells may hinder a secondary response, potentially explaining the reduction observed in all lymphocyte subpopulations, except for B lymphocytes, in the third determination. Despite this, B lymphocytes maintain similar levels to those observed in the first determination.

Furthermore, a decrease in immunoglobulin production is evident in the fourth determination, accompanied by a reduction in all lymphocyte subpopulations. This highlights the potential necessity for a booster dose. Interestingly, the fifth determination demonstrates a secondary cellular response following the administration of the third dose, leading to the restoration of all lymphocyte subpopulations and antibody production to levels comparable to those observed in the first determination. This suggests that the immune response to the third dose is significantly more efficient, involving both cellular and humoral responses, which occur simultaneously and complementarily.

In the secondary response, upon activation of T lymphocytes, there is subsequent promotion of B lymphocyte activation, resulting in a significant increase in IgG production. This phenomenon can explain the positive correlation observed between IgG and total T lymphocytes, as well as T cytotoxic lymphocytes, and the positive tendency observed with T helper lymphocytes. Ultimately, the administration of multiple vaccine doses facilitates a more robust secondary response not only by increasing B memory cells but also by promoting their differentiation into plasmatic cells, which secrete high-affinity IgG.

In our age analysis, three distinct age groups were established, considering the potential deterioration of immune system function with aging, which could manifest in varying immune responses to vaccination. Initially, our analysis evinced a negative association concerning humoral response, as median IgG levels were significantly higher in the younger group following the first dose administration. This finding is consistent with existing literature [6,9]. Nevertheless, in subsequent determinations, this difference was no longer statistically significant. It is important to note that in our study there is less representation of elderly people, given that the subjects included are active workers. Additionally, our study did not identify statistically significant differences in lymphocyte subpopulations between age groups.

An important limitation of the study is the absence of a whole blood sample before vaccination, which could have provided baseline lymphocyte subpopulations for comparison across all determinations. However, it is likely that all subjects included in the study had similar basal lymphocyte counts, as they were healthy individuals with no prior history of SARS-CoV-2 infection. Additionally, the stimulation of T and B memory lymphocytes with SARS-CoV-2 pools of peptides was not carried out due to the significant workload at the time. Finally, the decrease in sample size throughout the study due to loss of follow-up, as well as the need for rapid vaccination during continuous COVID-19 waves, represents another limitation. Although the sample size of the fourth determination does not allow us to draw any conclusions, we have included it to observe the trend of the humoral response to the vaccine over time.

## Conclusions

In summary, while the immune response to the vaccine is remarkable, it diminishes over time. Nevertheless, cellular immunity maintenance could potentially offer long-lasting protection against SARS-CoV-2 infection. This supports the adoption of a strategy similar to that of influenza vaccination, with seasonal vaccinations using the most recent variants. Emerging data suggest that cellular immune protection induced by vaccination may not be greatly impacted by new strains. Nevertheless, further studies are needed to gain a better understanding of the long-term immune response to SARS-CoV-2 vaccination and to determine the optimal immunization strategy.
